# Morphoelasticity in the development of brown alga *Ectocarpus siliculosus*: from cell rounding to branching

**DOI:** 10.1098/rsif.2016.0596

**Published:** 2017-02-22

**Authors:** Fei Jia, Martine Ben Amar, Bernard Billoud, Bénédicte Charrier

**Affiliations:** 1 School of Manufacturing Science and Engineering, Southwest University of Science and Technology, Sichuan 621010, People's Republic of China; 2 Laboratoire de Physique Statistique, Ecole Normale Supérieure, PSL Research University, Sorbonne Universités UPMC Univ Paris 06, CNRS, 24 rue Lhomond, 75005 Paris, France; 3 Institut Universitaire de Cancérologie, Faculté de médecine, Université Pierre et Marie Curie-Paris 6, 91 Bd de l'Hôpital, 75013 Paris, France; 4 UMR8227 CNRS-UPMC, Station Biologique, Place George Teissier, 29680 Roscoff, France

**Keywords:** growth, morphogenesis, cell division, seaweed, bioelasticity, poroelasticity

## Abstract

A biomechanical model is proposed for the growth of the brown alga *Ectocarpus siliculosus*. Featuring ramified uniseriate filaments, this alga has two modes of growth: apical growth and intercalary growth with branching. Apical growth occurs upon the mitosis of a young cell at one extremity and leads to a new tip cell followed by a cylindrical cell, whereas branching mainly occurs when a cylindrical cell becomes rounded and swells, forming a spherical cell. Given the continuous interplay between cell growth and swelling, a poroelastic model combining osmotic pressure and volumetric growth is considered for the whole cell, cytoplasm and cell wall. The model recovers the morphogenetic transformations of mature cells: transformation of a cylindrical shape into spherical shape with a volumetric increase, and then lateral branching. Our simulations show that the poro-elastic model, including the Mooney–Rivlin approach for hyper-elastic materials, can correctly reproduce the observations. In particular, branching appears to be a plasticity effect due to the high level of tension created after the increase in volume of mature cells.

## 1. Introduction

Morphoelasticity, the mathematical modelling of biological growth, is a fast-developing area in embryogenesis and plant morphogenesis. From simple growth laws, it can quantitatively explain the shapes observed in plants [1,2], algae [3,4], fungi and pollen tubes [5,6] and organs [7,8]. Its domain of validity applies to soft tissues, treated at the mesoscopic scale, hence larger than individual cells. However, even at the level of an individual cell, during mitosis in eukaryotic cells, mechanical forces play an important role [9,10] and can explain how cells divide. In this study, we propose a biomechanical model to explain key events in the development of the multicellular brown alga *Ectocarpus siliculosus* occurring at the cellular level which, by multiplication and competition, ultimately leads to the overall shape of this alga.

**Figure 1.**
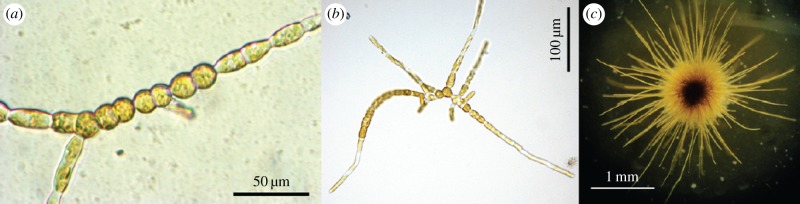
Developmental pattern of *Ectocarpus siliculosus*. (*a*) A 15-day-old sporophyte with few branching events; (*b*) a 20-day-old sporophyte after iteration of branching; and (*c*) a four-week-old adult sphorophyte. (Online version in colour.)

Brown algae have been less studied in the past compared with other algae (green, red or yellow-green). They strongly differ from land plants or green algae. For example, brown algae do not have microtubule cortex below the cell membrane and this cortex is known to play an essential role in the cellular morphogenesis of plants (because it controls the deposition of new material) [11–13]. From a phylogenetical viewpoint, brown algae are distinct from other multicellular lineages [14], and provide the opportunity to discover new developmental organization, unknown until now. Among brown algae, *E. siliculosus* is a model biological system for its ecological occurrence, and also as a genetic model [14,15] for all brown algae. Made of tufts of a few centimetres (figure 1), *E. siliculosus* exhibits a uniseriate filament tree structure. This simple morphology makes *Ectocarpus* an ideal candidate for studying the morphogenesis of brown algae. Moreover, *Ectocarpus* has a tremendous advantage that makes it a good model system: due to its uniseriate filament structure, each cell is in contact with the external environment, i.e. the water, simplifying experimentations and observations.

**Figure 2.**
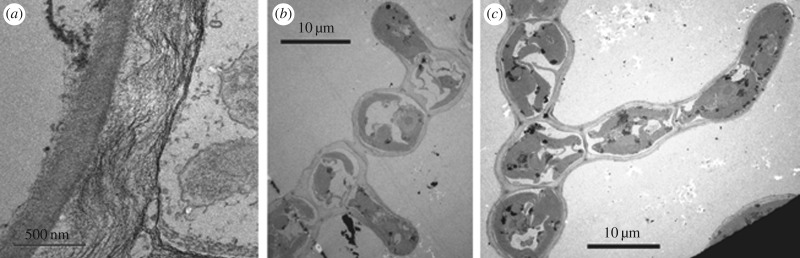
Electron microscopy images of *Ectocarpus siliculosus* cells. (*a*) Wall of a cylindrical cell; (*b*) rounding and branching events on neighbouring mature cells with initiation of mitosis; and (*c*) development of the branched filament by successive mitosis. Copyright Sophie Le Panse and Bénédicte Charrier, Station Biologique de Roscoff (France).

At early stages, *E. siliculosus* develops mostly by apical elongation of its filaments. Mitosis takes place perpendicularly to the axis of the apical cell, providing new cells after apical cell elongation, both events ensuring most of the filament growth. Some rare divisions take place in the middle of the filament and involve mature cells. Some of them give rise to a new filament, growing relatively perpendicularly to the main axis (figure 2). Since most new cells originate at the tip, their shapes are elongated and cylindrical, contrary to the mature cells at the centre of the filament which are spherical. Mature cells differentiate by ‘swelling’, i.e. changing from cylindrical to quasi-spherical shapes, then initiate the branching event at the origin of a new filament. Their volume increases during this shape transformation. The biological function of this cell differentiation in *Ectocarpus*, which leads to a change in shape, is not known. Rounding perhaps may afford better resistance to environmental variations.

Apical growth has been extensively investigated for diverse types of natural structures such as pollen tubes, fungi and root hairs [16,17]. Usually modelling is restricted to the tip of an infinite cell and growth is controlled by turgor pressure, which is high in these systems. Here, we address the role of physical forces in the transition of a cylindrical cell into a spherical one, and assess how this process can result in branching. We used a purely biomechanical model of growth and chose the poroelastic formalism, because it allows coupling of the osmotic pressure with the stresses induced simultaneously in the cell wall and in the cytoplasm. Elastic constants for the cell wall, necessary to reproduce the observations, have been measured^1^; unknown values for the soft interior were estimated or varied in our simulations. We tested two models of finite elasticity, the neo-Hookean (n-H) and the Mooney–Rivlin (M-R) models [19,20], and we performed the simulations using FEBio software.^2^ With this framework, we show that cell growth, combined with an imposed pressure, explains the morphological changes observed at the cellular level. In addition, branching appears to be the consequence of the accumulation of stresses in the cell wall, exceeding the stability limit and modifying the growth rate locally.

The paper is organized as follows: §2 gives a description of the cylindrical cell, cytoplasm and cell wall. §3 is devoted to the poroelastic model and details the two hyper-elastic models treated in the simulations. §4 focuses on the rounding of a cylindrical cell and §5 on the branching. Finally, the conclusion, §6, gives suggestions for future work on multiple branching events. Appendix A provides more details on the poroelastic model.

## 2. Morphology of *Ectocarpus siliculosus*: from cells to organism

Structural information on cell composition is necessary for morphogenetic studies, especially for living species which are protected by cell walls that are stiffer than the soft interior of the cell, such as in plants, bacteria and algae. As in plants, the cellular structure of *E. siliculosus* involves a cytoplasm, bounded by the plasma membrane and surrounded by a cell wall. The cytoplasm contains organelles such as the nucleus, the chloroplastic endoplasmic reticulum and envelope, mitochondria, lamellae and, in the centre, a vacuole contained in a membrane called the tonoplast (see fig. 4 of [15]). Half of the cytoplasmic volume is occupied by the giant ribbon-shaped chloroplast [15], the content of which is mainly made of lipids (thylakoids) and proteins (photosystems). The details of such complex inhomogeneous structures cannot be included in a biomechanical treatment, but one can keep in mind that the interior of the cell has a dual structure: a soft gelatinous substance and a liquid. The gel itself, involving lipid vesicles, proteins and organelles, has some stiffness, even if its value is not known presently.

Contrary to animal cells, the outer layer is surrounded by a cell wall: the lateral sides of the wall in contact with the environment (i.e the water) and the transversal walls that separate two neighbouring cells. For *E. siliculosus*, studies concerning wall composition generally focus on a phylogenetic perspective [21]. Like plants, this alga produces cellulose, but probably in a smaller proportion [22]. The main cell wall component is an anionic polysaccharide called alginate. However, the arrangement of the polymers inside the cell is not known, as only hypothetical models have been proposed in 1986 [23] and in 2014 [22]. More recently, however, a very detailed study [24] combining electron microscopy, electron tomography and chemical treatments revealed the full structure of *E. siliculosus* cell walls, both for the lateral and transversal walls. The chemical treatments in [24] confirm the composition of the walls. By changing the concentration of Ca^2+^ and adding sorbitol, it is possible to disintegrate the walls, proving that fibrils are made of alginate–calcium fibrous gels. Electron microscopy demonstrates that the cell walls have a multi-layer structure in which the alginate fibrils are not uniformly distributed and ordered. In fact, the lateral wall of an upright filament studied in [24] has three sub-layers, of approximately the same thickness, contrary to the transversal wall made of only two layers, and contrary to prostrate filaments which also exhibit two sub-layers (figure 2*a*). Each sub-layer is characterized by fibril organization. In the innermost layer, parallel and in close vicinity to the plasma membrane, the fibrils are denser and more ordered, aligned in the same direction as the plasma membrane, as shown in figure 2*a*. They become looser in the intermediate sub-layer and the alignment is weakened and even disappears in the outermost layer.

The electro-tomography technique identifies junctions between fibrils and quantifies their number: here again, more junctions are present in the innermost layer. These junctions may make the wall stiffer if the fibrils are linked together, a simple rule giving a Young's modulus proportional to the number of junctions per unit volume [25]. The inter-dependence between the fibrils of alginate and the cellulose micro-fibrils has not been fully elucidated, but it seems that cellulose micro-fibrils have no specific orientation. This is confirmed by transmission spectroscopy experiments as shown in figure 2*a*. This information on the anisotropy and inhomogeneity of the wall architecture is crucial for the modelling of the rounding and branching of mature cells. Clearly, to the best of our knowledge, the cells of this alga (interior and wall) cannot be considered as simple balloons under pressure, even in a crude approach. Unfortunately, to the best of our knowledge, there are no similar studies on apical (tip) cells, and we limit our work to rounding and branching.

## 3. The model

Because the life of an alga obviously takes place in water, the structure of the cells will depend on the surrounding water which is either the sea, freshwater or any nutrient bath in a laboratory vessel. The mechanical and chemical stresses these algae will undergo are diverse and likely change the cell wall composition. For this reason, we chose the poroelastic model which offers the possibility to include a mixture of materials: dry elastic compounds, a fluid compound and a solute responsible for the osmotic pressure, which may vary according to the cell environment [26,27].

### 3.1. From structural composition to biomechanical constant evaluation

Let us first discuss the dry compound since an important question for modelling involves the elastic behaviour of each compartment, once the cell structure has been established. However, the experimental mechanical measurements on *E. siliculosus* are uncommon. In [18], it is mentioned that the algal cell wall has a stiffness of between 1 and 100 MPa, but a more precise estimation of the Young's modulus *E*_c_ of the cell wall rather indicates 22 MPa, using preliminary atomic force microscopy experiments on prostrate filaments (see endnote 1). Theoretically, recent models have attempted to evaluate the elastic properties of fibre networks [28] from the microscopic structure, but the answer depends not only on the characteristics of one filament (its geometry and intrinsic bending modulus), but also on the matrix and the cross linkers, which here seem to be the cellulose micro-fibrils. A naive estimation of 1% of cellulose micro-fibrils, each of them having a Young's modulus of around 100 GPa [29], gives a stiffness of 1 GPa for the whole wall, which is of course exaggerated by a factor of order 50. For alginate hydrogels, the results also depend on concentration, but for a few per cent, stiffness is roughly 0.1 MPa (or less). This value is much lower than the estimated experimental value and also much softer than the cellulose stiffness estimation. Therefore, it is likely that the stiffness of the cell wall is dominated by cellulose micro-fibrils which are relatively disorganized and disordered, although mostly oriented perpendicularly to the alginate fibres, as demonstrated in [24]. The situation of the cell interior is even more complicated in the absence of any measurement. Therefore, in the following, we will vary the Young's modulus *E*_*s*_ between 0.1 and 5 MPa. In addition, we must keep in mind that these estimations only concern the ‘dry’ part or the solid component of the cell, which also contains water and solutes.

### 3.2. Our poroelastic model with three compounds

Our model will involve two different connected materials, both of them made of a mixture of three compounds: dry matter, fluid matter and a solute, located inside the cell at the source of osmotic pressure. Exchanges across the cell wall are possible for the fluid, but not for the solute. The poroelastic model allows these exchanges with the environment such as, for example, the outer reservoir of the cell or the sea whose detailed composition can be found in [30]. Considering the initial volume of the sample as the unit of volume, we call 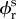, the solid volume fraction of the mixture (compared with the unit of volume), then the fluid volume fraction becomes 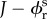 once the solute volume fraction is neglected. Since *J* concerns the whole mixture, in the absence of growth, *J* = Det(**F**), where **F** is the elastic deformation gradient of the whole mixture. In the following, we use the convention of bold letters for tensors. If *c*_r_ is the number of moles per unit volume of the solute, *c*_e_ the osmolarity of the external environment (the sea for example), then osmotic pressure is, for a dilute solution, according to the laws of thermodynamics3.1
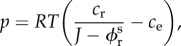
where *R* is the universal gas constant: *R* = 8.314 × 10^−6^ MPa (nmol K)^−1^, *T* the absolute temperature: *T* = 298 K. To ensure that the initial cylindrical configuration is a stress-free reference configuration, we impose 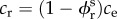 in the initial state prior to growth. Then we increase the outer tension −*p*_e_ (obtained from equation (3.1)) to reach the desired osmotic pressure and we get a configuration that is obviously not stress free. Considering the case of biological growth and no swelling, all the constituents of the cell will vary identically with the same factor (this hypothesis is not mandatory, but is reasonable). Then *c*_r_ and *ϕ*_r_ will be multiplied by the same factor of proportionality to represent biological growth [31,32]. Mechanical stresses are induced by growth under the constraints of the boundary conditions, at the border between the wall and the cell interior, and the wall and the sea. In addition, a strong constraint originates from the limited possibility of extension of the growing cell because it is confined by its two neighbours. Owing to the rather strong deformation of the cell during the rounding event, which approximatively doubles the cell volume, mechanical stresses are evaluated by finite elasticity for soft materials [19,20]. Although the rounding is a dynamic process, it is very slow compared with any mechanical time-scale. Therefore, from the viewpoint of elasticity, the cell shape results from an equilibrium between the osmotic pressure and the boundary constraints, at a given volume. Thus, the cell elasticity model (the wall and the cell interior) has tremendous importance and must be considered in detail. Hence, Cauchy stress [19,20] is related to the elastic solid stress according to3.2
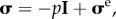
where ***σ***^e^ is the stress derived from the hyper-elastic models and *p* is given by equation (3.1). The complete set of partial differential equations is detailed in [31,32] and in the FEBio manual (see endnote 2). Applying the equilibrium equation 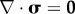 and the boundary conditions helps to solve the unknown *J* which gives the final volume of the cell. A simple example is given in appendix A. For the cell wall, different initial data can be chosen. Many constitutive laws for biological tissues have been introduced, more or less simple in terms of the fundamental invariants *I*_*j*_ of strain. The most common are the n-H and the M-R models [19]. Because the cell interior is gelatinous, the n-H model is well adapted. However, for the cell wall, a more sophisticated representation may be necessary. Superposed on both elastic models (n-H or M-R), we can include fibres in the cell walls, but from the modelling viewpoint, disorganized fibres (cellulose micro-fibrils) without a well-defined orientation simply make the cell wall stiffer [33,34]. We do not include the elasticity of fibres (except for numerical tests). However, they are incorporated in a coarse-grained approach in n-H and M-R models. Of course, neglecting the orientation of the fibres is justified for growth of cylindrical cells but maybe not for the apical cell [35]. It is worthwhile to mention that including the fibres in the model increases the number of invariants [19,20] as well as the number of unknown parameters because unfortunately there is little experimental data for *Ectocarpus*. Let us first explain the hyper-elasticity model chosen for the cell.

### 3.3. The neo-Hookean elasticity for the solid component of the poroelastic model

The compressible n-H model is the one most encountered in biomechanics. Based on the entropic elasticity of polymer solutions [36], it has a nonlinear stress–strain behaviour and it reduces to the classical linear elasticity for small strains. The hyper-elastic energy density per unit volume *W*_n-H_ requires two coefficients:3.3
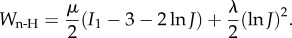
*I*_1_ is the first invariant of the right Cauchy–Green deformation tensor: *I*_1_ = Tr(**F^T^ F**). *λ* and *μ* are the Lamé parameters defined in linear elasticity and related to the Young's modulus *E* and Poisson ratio *ν* via the relationships [37]3.4
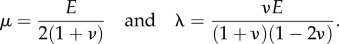


### 3.4. The Mooney–Rivlin model

The M-R model is sometimes preferred: due to an additive parameter, it allows a more flexible representation of nonlinearities at large deformations. It reads3.5
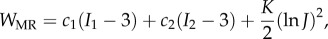
where *c*_1_ and *c*_2_ are the M-R coefficients and *I*_2_ is the second invariant of the deviatoric right Cauchy–Green deformation tensor: 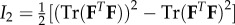. The coefficient *K* is the bulk modulus or a penalty parameter for volume increase. It has the same significance as linear elasticity [37]. For small strains, we can relate *c*_1_ and *c*_2_ to the shear modulus *μ*, introduced in equation (3.4), and obtain3.6
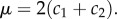


## 4. From cylindrical to spherical cells

**Figure 3.**
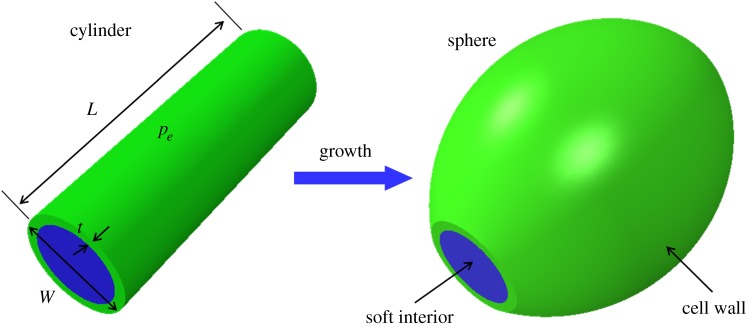
Geometry of the cell.

Differentiation occurs in mature cylindrical cells located at some distance from the apical cell. The process of transforming the initial cylindrical shape into a quasi-spherical one (figure 3) occurs via a significant increase of volume, approximately doubling the initial volume, calculated from the cell dimensions reported in [38]. Differentiation is a much more delicate problem compared with the growth of a sphere studied previously [39,40]. The cylinder to sphere transformation requires that the two transversal cell walls, frontiers between two neighbouring cells, do not move and growth is constrained by the adjacent cells. To explain this geometrical transformation, the combined action of growth and of external tension is required. The osmotic pressure due to the presence of the solute plays this role. Thus, the cell is initially modelled as a cylinder made of a thin external elastic wall and a soft gelatinous interior (figure 3), with length *L* set to *L* = 30 *µ*m, *W* the initial diameter set to *W* = 10 *µ*m, these two values being given in [38], and a thickness *t* chosen as *t* = 1 *µ*m (estimated from figure 2*a*). These sizes are different from those we can extract from [24], especially the diameter which is closer to 25 *µ*m. For convenience, the osmotic pressure is represented by a negative pressure − *p*_e_ (or tension) applied to the outside surface of the cell wall that we vary. The internal cell pressure, 3.34 MPa, according to [41], is about 1 MPa higher than the external medium (the sea water being at 2.3 MPa, according to [30]). The period between the increase in external tension and the growth process is not crucial for computations, and can be considered either instantaneous or long-lasting. In practice, for simplicity, the numerical simulation of cell rounding was decomposed into two steps. The first step consisted of applying linearly increasing pressure; the second step involved coupling the imposed pressure with the increase in cell volume. In the numerical simulations, we chose values in the vicinity of the experimental ones, but we also simulated several examples to show the competition between growth and osmotic pressure during the shape transition, varying the elasticity of both cell components: the wall and the cell interior.

### 4.1. Results with the Neo-Hookean hyper-elasticity

**Figure 4.**
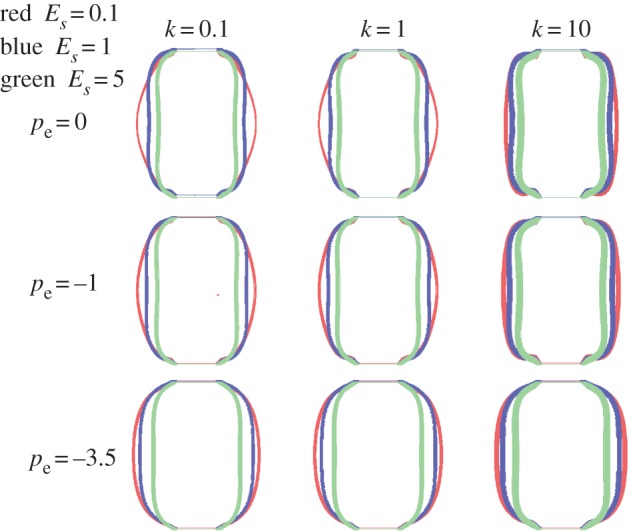
Final cell shapes when growth proceeds according to the n-H model for both the cell wall and the cell interior. The wall Young's modulus is fixed to the experimental value of 22 MPa while the cell interior Young's modulus *E*_*s*_ varies from 0.1, 1 to 5 (red, blue and green lines, respectively). Lines indicate the cell contour during growth, the line thickness represents the numerical results. Note the rounding that occurs with time and a slight decrease in thickness. The wall tension −*p*_e_ (opposite of pressure) is applied to the external cell wall with varying amplitudes from top to bottom.

**Figure 5.**
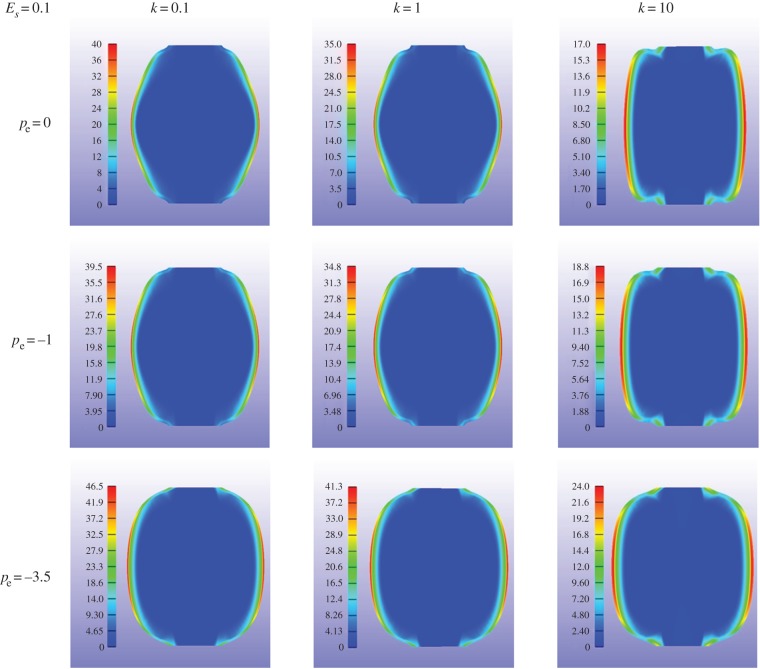
Final cell shapes, the interior Young's modulus being *E*_*s*_ = 0.1 MPa, in the n-H approximation (for the definition, see equation (3.3)). Tension (opposite of pressure) is applied with varying amplitude. Colours in the cell indicate the level of the von Mises stresses (see colour scales) inside the cell wall.

This model was chosen both for the cell wall and the gelatinous cell interior, but with different values. The elastic properties and the growth of the cell are listed in table 1. All parameters were chosen to gradually represent growth from an initially cylindrical shape to a rounding shape. Thus, *ϕ*_r_^s^ and *c*_r_ increased over computational time while *c*_e_ was kept constant. Much larger than the cell wall, the soft interior contributes more in the change of shape and parameters were chosen to be as close as possible to the experiments. The growth ratio of the cell wall is controlled by *k* (see appendix A for the role of *k*). Increasing the magnitude of *k* induces cell growth. Three different values of *k* were selected (0.1, 1, 10) and three values for pressure (0 MPa, −1 MPa, −3.5 MPa). The value of the cell interior Young's modulus *E*_*s*_ also varied, but was small compared with the cell wall *E*_c_ = 22 MPa, deduced from the experiment (see endnote 1). The corresponding results, with different values of the Young's modulus of the cell interior (*E*_*s*_ = 0.1 MPa, 1 MPa, 5 MPa) are given in each panel of figure 4. The cell wall is represented in colour with a thickness calculated by the model. Independently of the over-pressure value, a soft interior favours rounding and even under growth conditions, a more rigid cell interior favours a cylindrical shape. Pressure also favours rounding; however, when growth increases (with the parameter *k*), the thickening of the cell wall slightly inhibits rounding. Our calculation does not show obvious thickness variation along the cell wall. From left to right in every row of figure 4, the cell wall becomes thicker under the same external pressure. When thickness increases, the cell wall becomes stiffer and is more difficult to push out from the interior. Hence, the shape becomes a puffy cylinder at the end of each row. From top to bottom in each column, pressure increases the volume of the cell and makes it rounder. Moreover, the thickness of the cell wall becomes more uniform. The results for *E*_*s*_ = 1 MPa and 5 MPa in figure 4 (blue and green lines) illustrate that cell volume decreases with increased modulus *E*_*s*_. This is due to the coupling effects, equation (3.2), between the volume change *J* and the elastic properties of the matrix material. The final shapes are more like a peanut than a sphere under low pressure. This shape is similar to the symmetrical buckling that occurs in a bilayer cylinder under growth [42]. The von Mises stress distribution in the cell wall for *E*_*s*_ = 0.1 MPa is indicated in each panel of figure 5. This case corresponds to figure 4, red lines. The cell wall is coloured according to the legend bar on left. The von Mises stress is a scalar stress value that can be computed from the Cauchy stress tensor4.1
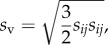
where *s*_*ij*_ is the component of the stress deviator tensor 
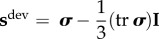
. It is an indicator of the level of stress generated by the deformation. For all cases, the highest stress value is located in the centre of the cell wall, which shows that this part can be elastically weakened, thus allowing branching under pressure. This fact justifies the choice of the location of the branching initiation at the centre, as shown in the next section.

**Table 1.** Cellular parameters.

	*c*_r_(nmol mm^−3^)	*c*_e_(nmol mm^−3^)	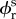	*E*(MPa)	*ν*
cell wall	*k*[90 − 810]	*k* × 100	[0.1 − 0.9]	22	0.4
soft interior	[900 − 8100]	1000	[0.1 − 0.9]	*E*_*s*_	0.4

### 4.2. Results with the Mooney–Rivlin hyper-elasticity

**Figure 6.**
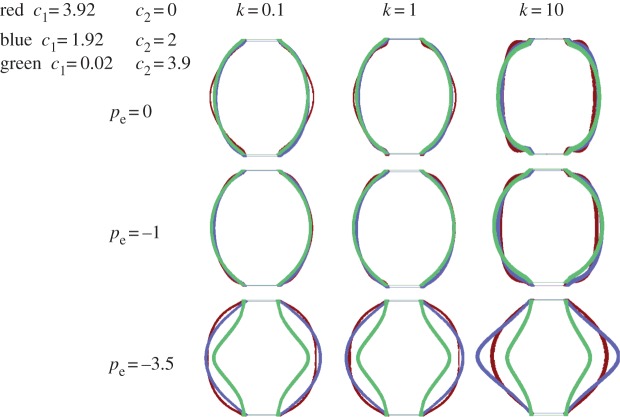
Cell shapes according to the M-R model. Definitions of the coefficients are given in equation (3.3). Tension (i.e. opposite of pressure) is applied with varying amplitude.

The following correspondence can be achieved between these coefficients and the measured Young's modulus *E*_c_ according to the relationships of equation (3.6):4.2
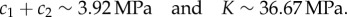
From equation (4.2), we assign three values to the couple (*c*_1_, *c*_2_). For the first case, *c*_1_ = 3.92, *c*_2_ = 0, the M-R model reduces to the n-H constitutive model, close to the limit of incompressibility. The final shapes in figure 6 (shown in red) are similar to the ones of figure 4 (shown in red) for n-H materials, but the cells are much rounder especially when the pressure increases to −3.5 MPa. Unsurprisingly, pressure always makes the cell rounder. Although its experimental value is relatively low compared to the cell wall, it has a noticeable effect. When growth is greater, the calculation cannot always converge. In figure 6 (shown in blue) *c*_1_ = 1.92 and *c*_2_ = 2, one important feature is that the thickness of the cell wall becomes uniform for each shape. The shapes are generally spherical except the last one with more growth and high pressure. Finally, in the third case, *c*_1_ = 0.02, *c*_2_ = 3.9, the shape is similar to the second case when the pressure is −1 MPa and 0 MPa. Because of the well-known singularity of the M-R model, when *c*_2_ is negative, convergence also becomes difficult in this range of parameters and growth is limited. As above, the M-R model gives a variety of different final shapes, for different parameter values. Regardless of the elastic model used, our simulations do not show wrinkling instabilities characteristic of cylinder geometry [42–44].

## 5. Branching

**Figure 7.**
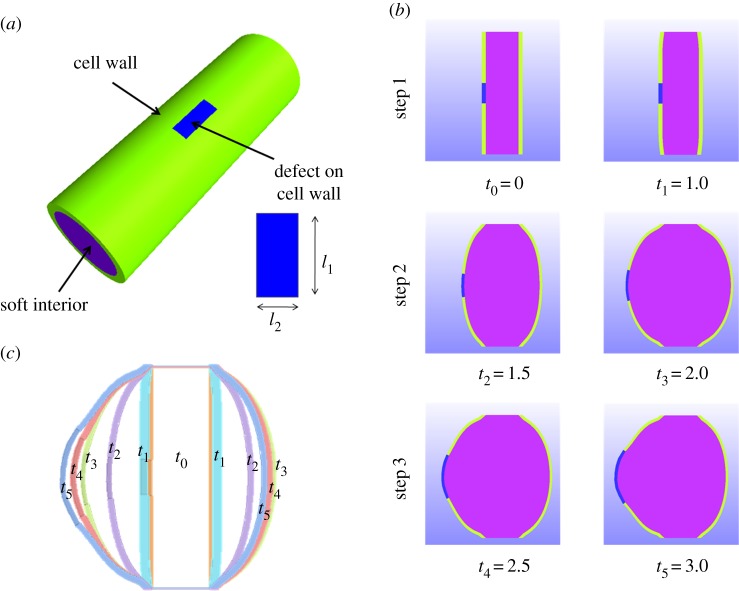
(*a*) Geometry of the cell with a plastic zone located in mid-position. (*b*) Change in the cell shape in the three steps of growth as described in §5. Note the right–left asymmetry as growth proceeds. (*c*) Change in the cell wall over time.

Filament branching occurs mainly on round cells in *Ectocarpus* [38]. Therefore, we attempted to model the emergence of a branch once the rounding process is finished. From the current observation, we assumed that branching corresponds to a bulge in the orthogonal direction of the primary filament. Subsequent apical growth of this bulge generates a new cell through mitosis, and re-iteration of the growth steps leads to the formation of a secondary filament. For a better understanding of this branching phenomenon, we modified our previous model and introduced plasticity at a precise location on the external surface of the cell wall that corresponds to a high tensile state. This plasticity induces an increase in the local growth rate, due to the excess tension, according to the law of homeostasis, or similarly, to Lockhart's law for plants [45]. Both laws suggest the existence of a tensional threshold to induce proliferation. In the case of plants, in the original paper of Lockhart [45], growth is represented by an irreversible extension of the cell wall which occurs when the stresses, induced by turgor pressure acting on the cell wall, exceed a given threshold called yield stress. In the case of a cylindrical cell, this law predicts that the relative rate of length increase is directly proportional to the difference between the tension inside the wall and a critical value called the yield stress. For computational purposes, we cannot apply this law gradually. We simplify the model by a rectangular plastic zone with the highest growth rate. Initially, this zone is located on one side, at the middle (figure 7*a*). Owing to growth, it changes its shape and makes a tip shown in figure 7*b*,*c*. This particular zone is at the origin of the branching event. All the parameters of the cell materials are listed in table 2. The whole simulation now consists of three different steps:

(1) Application of the pressure *p*_e_ = −1 MPa on the external surface of the cell wall; it is maintained in steps 2 and 3.
(2) Growth of the whole cell.
(3) Growth arrest except for the branched part.

**Table 2.** Parameters for the branching of an initially cylindrical cell.

	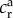	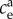	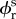	elastic parameters^b^
cell wall	[90 − 810]	100	[0.1 − 0.9]	*c*_1_ = 1.92; *c*_2_ = 2; *K* = 36.67
interior	[900 − 8100]	1000	[0.1 − 0.9]	*E*_*s*_ = 0.1; *ν* = 0.4
tip area	200[9.8 − 98 − 294]	2000	[0.02 − 0.2 − 0.6]	*c*_1_ = 1.92; *c*_2_ = 2; *K* = 36.67

^a^Values are in nmol mm^−3^. ^b^The Young's modulus *E*_*s*_ and Poisson's ratio *ν* for the neo-Hookean model are according to equation (3.3) and *c*_1_, *c*_2_ and *K* are the coefficients of the Mooney–Rivlin model according to equation (3.5).

**Figure 8.**
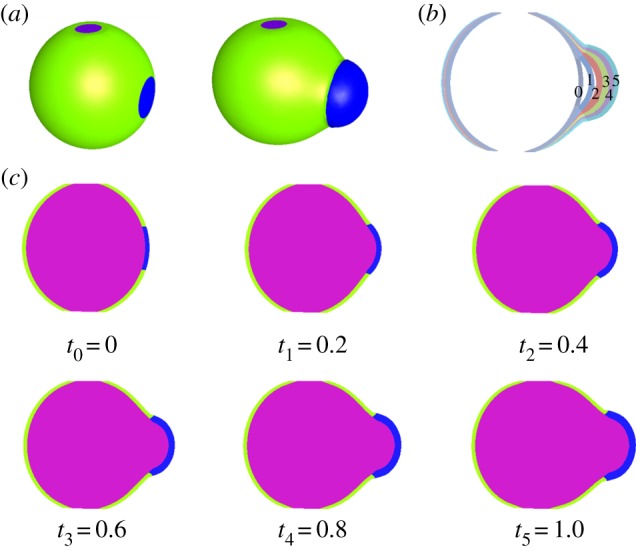
(*a*) Geometry of the cell with a plastic zone located in mid-position, (*b*) change in the cell wall over time and (*c*) change in the cell shape during growth.

The results for the cell shape are shown in figure 7*b* at six different times. At first time, the cell shape changes slowly under pressure, then becomes more rounded and relatively symmetrical and grows significantly as shown in step 2. In step 3, dissymmetry appears between the left and right sides of the cell and distortion increases on the left side where the plastic zone is located. Such asymmetry is surprising and unexpected: it is as if all the excess pressure concentrates in the bulge. It is also observed in our set of photographs and movies (not shown) and in the picture in figure 2*c*. Despite differences in the growth coefficients, the wall thickness remains relatively homogeneous with constant thickness. This is typical of the M-R model; the same simulation performed with the n-H model fails to give satisfactory results. In figure 7*c*, the change in the external wall over time is shown in the same graph. In this graph, the wall thickness corresponds to the simulations. For practical reasons, going from an initially cylindrical to a rounded cell, then to a distorted cell with a bulge requires introducing by hand a plastic zone in the computations, in a discontinuous manner due to software limitations. This plastic zone exists in living systems because mechanical forces automatically induce a local modification in the wall structure and also modify growth, as indicated by Lockhart's law [45]. Of course, these modifications occur gradually in nature, contrary to our simulations which introduce a discontinuous growth behaviour. Several geometries were tested for the plastic zone and it turns out that the quasi-rectangular one, which looks like the initial cylindrical geometry, was the most adequate. In order to test our simulations, we made another choice. We began with a quasi-spherical cellular shape, choosing a rounded cell shown in figure 8, as initial configuration. The height of the cell is *L* = 30 *µ*m, the diameter of the top and bottom ends is *D* = 10 *µ*m, the diameter of the central part is *D*_c_ = 30 *µ*m. The thickness of the cell wall is *t* = 1 *µ*m. This spherical shape looks like the final equilibrium state under growth and osmotic pressure, but here it is chosen as an initial stress-free configuration. Hence, the pressure is no longer applied, cell growth is reduced except in the circular plastic zone, of initial radius *R*_*f*_ = 3 *µ*m, located on one side. The whole simulation consists only of one step. All the parameters of the cell materials are listed in table 3. The results also give a correct explanation for branching. However, with these initial conditions, which mimic the usual case of an already round cell undergoing a branching event, the change in shape appears to be restricted to the plastic zone and its immediate vicinity. In particular, the opposite side of the cell is not flattened as it is when the defect in the cell wall is present at the time of rounding (cf. figures 7 and 8). From the viewpoint of elasticity, the two approaches are different, since the latter process eliminates the stresses accumulated by the rounding of the cylinder. The flattening on the opposite side of the bulge, experimentally observed (figure 2*b*), is of course in favour of the cylinder transformation treatment, but observation of branching events in our collection set also shows the second possibility. We conclude that the two scenarios occur in nature.

**Table 3.** Cellular parameters for the branching of a quasi-spherical cell.

	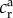	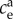	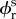	Elastic parameters^b^
cell wall	[0.9 − 8.1]	1	[0.1 − 0.9]	*c*_1_ = 1.92; *c*_2_ = 2; *K* = 36.67
interior	[4.5 − 40.5]	5	[0.1 − 0.9]	*E*_*s*_ = 0.1; *ν* = 0.4
tip area	1000[9 − 81]	10000	[0.1 − 0.9]	*c*_1_ = 1.92; *c*_2_ = 2; *K* = 36.67

^a^Values are in nmol mm^−3^. ^*b*^The Young's modulus *E*_*s*_ and Poisson's ratio *ν* for the neo-Hookean model are according to equation (3.3) and *c*_1_, *c*_2_ and *K* are the coefficients of the M-R model according to equation (3.5).

## 6. Conclusion

The poroelastic model with M-R hyper-elasticity gives a satisfactory representation of the key features occurring during the morphogenesis of *E. siliculosus*, a brown alga made of uniseriate filaments. Here, we focused on the change from a cylindrical to a spherical cell via an increase in volume and on branching. These cellular events concern mature cells located in the central half of the filament. The simulations, carried out with FEBio software (see endnote 2), give satisfactory information on cellular morphogenetic events for a range of realistic elastic parameters, even if this software does not have the flexibility required for mechano-transduction processes necessary for branching. We would have preferred to introduce the plastic zone continuously as a function of the local stress induced by growth and osmotic pressure. This study shows the importance of the coupling of biological growth and mechanical properties in cell morphology. Growth clearly induces residual stresses in the cell wall, initiating a symmetry breaking and bulging process, then a mitosis event as shown in the experiments (figure 2*c*). Future studies can explore the competition of branching events in neighbouring quasi-spherical cells as shown in figure 2*b*. Here, the cells are considered as isolated with interactions between neighbours limited to the inter-cellular junctions. However, neighbouring cells communicate via ion channels and the process of rounding is not completely independent. A previous model (cellular automaton Ectomat [46]) suggested that the rounding process depends only on the geometrical nature of the neighbouring cells: rounding will occur only if one neighbouring cell is already round. Hence, at least one cell must be round in the initial stages to allow the initiation of the series of cell shape transitions. Another future direction involves apical growth. Many theoretical models have been proposed in the recent past, concerning yeast, pollen tubes and fungi. With more biological information on the wall structure at the tip, these models can be good candidates for studying morphogenetic processes in *E. siliculosus* also.

## Endnotes

1 Charrier B *et al.* unpublished results obtained with atomic force microscopy, technique explained in [18].

2 FEBio software for nonlinear finite-element analysis in biomechanics and biophysics, developed at the University of Utah and Columbia University (free access).

## Appendix A

In this appendix, we explain the method used in FEBio software that we adapted to our study. Cell growth can be modelled by using equation (3.1) in §3.2 for fluid pressure and allowing the parameters  and *c*_r_ (normally constant) to increase over time as a result of biological growth. Since cell growth is often accompanied by cell division, and since daughter cells typically have the same solid and solute content as their parent cell, it is convenient to assume that  and *c*_r_ increase proportionally. To ensure that the initial configuration is a stress-free reference configuration, let  in the initial state prior to growth. The Cauchy stress for the cell growth model is

A 1

For instance, *c*_r_ = [900 − *ρ*900], , *c*_e_ = 1000 as shown in table 1 ([*a* − *ρa*] represents the interval variation). From equation (3.1), assuming isotropic free growth and isotropic shape, ***σ***^e^ is only a function of *J* which depends on the hyper-elastic model, as explained in §3.2. Defining *F*(*J*), such that ***σ***_e_ = *F*(*J*)**I**, we derive an equation for *J*

A 2

However, in our case, we have boundary conditions that generate a non-trivial shape for our cell. As mentioned in §3.2, the equilibrium equations are then

A 3

In addition, transverse walls are considered as rigid and there is no displacement. The determination of *J*_*s*_ can only be done by using finite-element numerical software. It is not a constant and only the final calculation gives us an estimation of cell growth. A factor *k* can also be introduced in the pressure on the left-hand side of equation (A 2). It modifies the growth property of the cell wall compared with the interior or to the plastic zone compared with the wall, in the case of different materials. As shown in figure 5, increasing *k* makes the wall more rigid because *k* = 10 gives a growing cell which remains roughly cylindrical.
